# First pan-specific vNAR against human TGF-β as a potential therapeutic application: in silico modeling assessment

**DOI:** 10.1038/s41598-023-30623-x

**Published:** 2023-03-03

**Authors:** Mirna Burciaga-Flores, Ana Laura Márquez-Aguirre, Salvador Dueñas, Jahaziel Gasperin-Bulbarela, Alexei F. Licea-Navarro, Tanya A. Camacho-Villegas

**Affiliations:** 1 Unidad de Biotecnología Médica y Farmacéutica, Centro de Investigación y Asistencia en Tecnología y Diseño del Estado de Jalisco (CIATEJ), Guadalajara, Jalisco México; 2 División de Biología Experimental y Aplicada, Centro de Investigación y Educación Superior de Ensenada (CICESE), Ensenada, B.C México; 3 CONACYT - Unidad de Biotecnología Médica y Farmacéutica, Centro de Investigación y Asistencia en Tecnología y Diseño del Estado de Jalisco (CIATEJ), Guadalajara, Jalisco México

**Keywords:** Applied immunology, Immunotherapy, Immunology

## Abstract

Immunotherapies based on antibody fragments have been developed and applied to human diseases, describing novel antibody formats. The vNAR domains have a potential therapeutic use related to their unique properties. This work used a non-immunized *Heterodontus francisci* shark library to obtain a vNAR with recognition of TGF-β isoforms. The isolated vNAR T1 selected by phage display demonstrated binding of the vNAR T1 to TGF-β isoforms (-β1, -β2, -β3) by direct ELISA assay. These results are supported by using for the first time the Single-Cycle kinetics (SCK) method for Surface plasmon resonance (SPR) analysis for a vNAR. Also, the vNAR T1 shows an equilibrium dissociation constant (*K*_*D*_) of 9.61 × 10^–8^ M against rhTGF-β1. Furthermore, the molecular docking analysis revealed that the vNAR T1 interacts with amino acid residues of TGF-β1, which are essential for interaction with type I and II TGF-β receptors. The vNAR T1 is the first pan-specific shark domain reported against the three hTGF-β isoforms and a potential alternative to overcome the challenges related to the modulation of TGF-β levels implicated in several human diseases such as fibrosis, cancer, and COVID-19.

## Introduction

In humans there are three Transforming Growth Factor beta isoforms (TGF-β1, TGF-β2, TGF-β3) as homodimer of 25 kDa with high sequence identity (~ 76%), similarity (86–91%)^1^, functions^2^, and canonical signaling pathways. The transmembrane receptors TGF-β type I (TβRI) and TGF-β type II (TβRII) recognize the TGF-β soluble homodimer. The binding of TGF-β/receptors lead to the activation of transcription factors, such as Smad’s or MAP kinases and Akt, that promotes the activation of diverse genes^3^. Nevertheless, the TGF-β1 isoform was described as the most prevalent and characterized, and its imbalance has been associated with human diseases^4^. TGF-β1 has proliferative and anti-proliferative properties depending on the microenvironment. In cancer, TGF-β1 favors the tumor progression by blocking immunological checkpoints and acting as an immunosuppressive cytokine^5^. TGF-β1 causes proliferation, angiogenesis, and excessive extracellular matrix (ECM) deposition in fibrosis leading to tissue damage^6^. Recently, researchers found a relationship between an increasing TGF-β serum concentrations and tissue damage in the brain, heart, and lungs in patients with the severe or persistent post-COVID syndrome (PPCS)^7–10^, stating the relevance of this cytokine and the urgency for new therapeutic options.

There are pharmacological blockade strategies against TGF-β based on using conventional monoclonal antibodies (mAbs) like Fresolimumab (GC1008, Genzyme/Sanofi), a pan-specific fully humanized IgG^11,12^. Small inhibitors of TGF-β receptor type I, such as Galunisertib monohydrate (LY2157299, Eli Lilly), or inhibitors of the activin receptor-like kinase 5 (ALK5), such as Vactosertib (EW-7197 or TEW-7197)^13,14^, or antagonist of the TGF-β type I (TβRI) and type II (TβRII) receptors, such as Losartan^15^. Other approaches use chimeric proteins, including the soluble extracellular domain of the TβRI and TβRII receptors, expressed as an immunoglobulin-Fc fusion protein (TβRII-Fc)^16–18^. Those pharmacological molecules focus on treating human diseases associated with the overexpression of TGF-β, such as chronic renal failure^19^, fibrosis^6^, and cancer^20^. Indeed, mAbs have been used as therapeutic agents, providing promising results in treating these diseases^21^. In that sense, single-domain antibodies (sdAb) have become more interesting for the biopharmaceutical industry because of their small molecular weight, thermostability, high affinity and avidity, and capacity to recognize and neutralize a variety of antigens, increased tissue penetration and refolding capacity^22–27^. This kind of antibody domain, isolated from camelids (vHH) or sharks and rays (vNAR), is part of the third generation of antibody-based therapeutic agents^28^, described as potentially more efficient than conventional mAbs^29,30^. The vNAR domains have been proposed as an attractive therapeutic and diagnostic alternative due to their features, mainly due to their small size, deep penetration into dense tissue^31–34^ and high affinity. There are vNARs previously described against human cytokines, i.e. as monomers; a vNARs anti-TNF-α demonstrated cytokine neutralization in an LPS murine model^35^ or formatted designs as dimers, trimers, or tetramers with improved affinity and extended lifetime circulation^36^. They also have a vNAR anti-VEGF165, demonstrating eye barrier penetration and angiogenesis decrease in the macula^37^. Nevertheless, to the best of our knowledge, is not previously been described a vNAR that recognizes the TGF-β cytokine. These previous reports demonstrated the potential impact of vNAR as a novel immunotherapeutic for human illnesses associated with cytokines recognition or neutralization. All vNARs mentioned above were isolated using the phage display technology, where the immobilized antigen was in solid support. An M13 bacteriophage library (generally produced in-house based on naïve or immune shark) panned against the cytokine^38^. In brief, the panning includes three of four repetition cycles of vNAR/cytokine binding incubation, washing steps that could increase each round, and a final elution of vNAR with cytokine binding potential.

Another future advantage of vNARs is the possibility of delivery via inhalation for treating diseases such as lung fibrosis, lung cancer, and severe COVID-19^26,31–34^. The delivery of vNARs using nebulizers is an attractive option because the delivered amount of the drug is concentrated in the lung minimizing the dosage volume, and their thermal stability^22^.

In human diseases such as fibrosis, cancer, and PPCS, the TGF-β cytokine is relevant. On the other hand, the vNARs domains are novel biomolecules for therapeutic or diagnosis usages. In the current work, we isolated and characterized one His-tagged vNAR domain; that recognizes all three recombinant human TGF-β soluble isoforms (rhTGF-β1-3). We describe the vNAR T1 as the first pan-specific shark domain against a cytokine. After three panning rounds of phage display, vNAR T1 was selected from a naïve shark library. The vNAR T1 domain has an extensive CDR3 (24 aa) that interacts with amino acids of the TGF-β cytokine isoforms, as demonstrated by molecular dynamics.

Interestingly, these identified amino acids are also recognized by the receptors TβRI and TβRII. The equilibrium dissociation constant (*K*_*D*_) for vNAR T1 was determined at 9.61 × 10^–8^ M using the superficial plasmon resonance equipment under the Single-Cycle kinetics (SCK) protocol. Furthermore, the specific amino acids of the cytokine that interact with the vNAR T1 are determined by in silico modeling. Also, the in silico affinity of vNAR T1 was determined for each rhTGF-β isoform by molecular dynamic, confirming that the vNAR interacts with the same amino acid as the natural receptor. These results imply that vNAR T1 can recognize all three TGF-β isoforms in silico and ELISA assays, making the vNAR T1 the first pan-specific vNAR domain with potential therapeutic applications.

## Results

### Selection of vNAR antibody from a non-immune library

**Figure 1:**
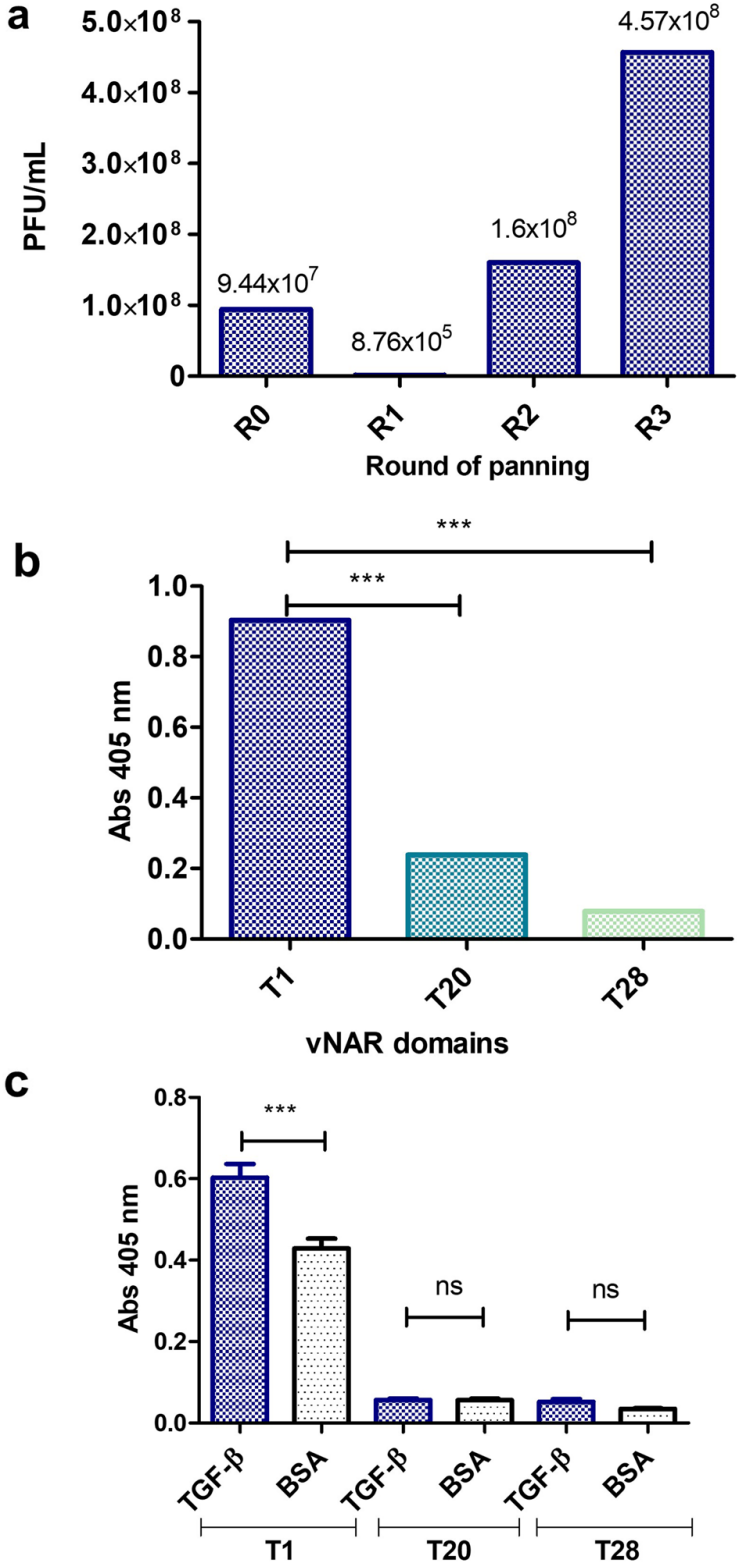
Isolation of anti-TGF-β vNAR. (**a**) Panning rounds with naïve library against rhTGF-β1, (**b**) Analysis of the periplasmic expression of vNAR domains, (**c**) Recognition ELISA assay of vNAR domains periplasmic extracts against rhTGF-β1. Error bars represent standard deviation (s. d.), n = 3. ***P < 0.001, **P < 0.01, and *P < 0.05.

**Figure 2:**
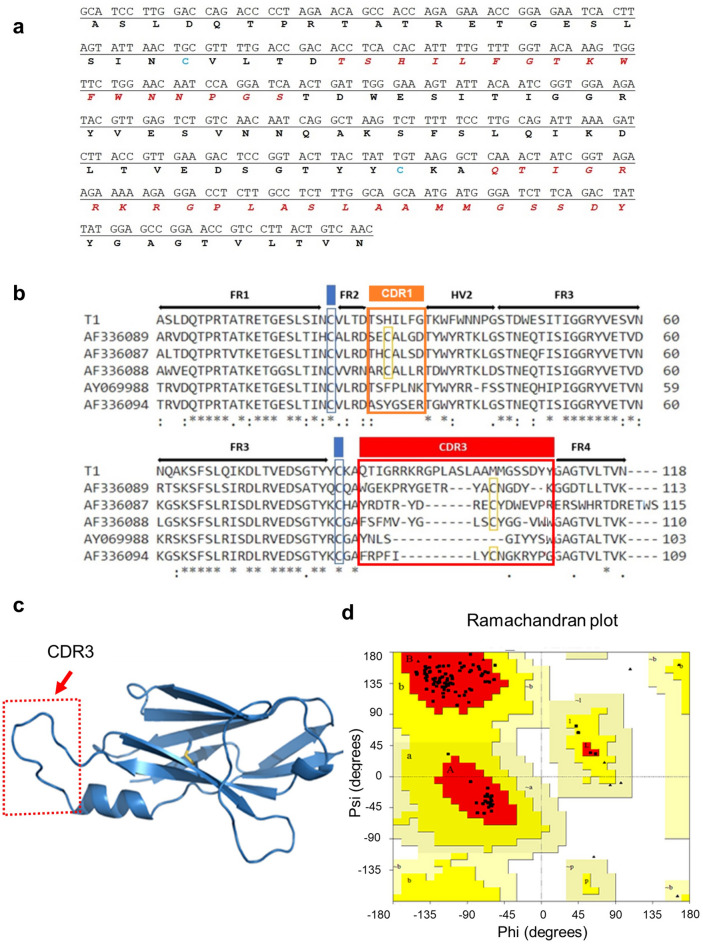
Nucleotide and aminoacidic sequences of the vNAR T1**.** (**a**) Nucleotide and aminoacidic sequences of vNAR T1. (**b**) Multiple Sequence Alignment (MSA) of vNAR T1 domain with other vNAR sequences (GenBank AF336089, AF336087; AF336088; AY069988; AF336094), showing a long CDR3 (24 aa) of the vNAR T1 with recognition capacity against rhTGF-β. The CDR1 region is in an orange box. The CDR3 region is in a red box. The canonical cysteine residues (amino acids: 22 and 83) in FR1 and FR3 regions (highlighted in blue). The non-canonical cysteine residues in CDR1 and CDR3 regions (highlighted in yellow). (**c**) Structural analysis of vNAR T1 shows that the CDR3 region acts as a hairpin with the binding capacity of TGF-β isoforms (CDR3 region in a red box). (**d**) Ramachandran plot of the model shows 99 residues (96.1%) in favored regions and 4 residues (3.9%) in additional regions.

After performing three rounds of panning by phage display, we obtained final titers of 4.5 × 10^8^ CFU/mL in *E. coli* ER2537 (Fig. 1a). Compared with BSA after each panning round, the phage pool with recognition capacity against rhTGF-β1 was increased. After a PCR screening of 32 isolated clones, three different vNAR domains were obtained: T1, T20, and T28. The plasmid and sequences were obtained from each clone to verify the open reading frame (ORF) and DNA sequence integrity of vNARs. Figure 1b shows the soluble periplasmic extract of the T1, T20, and T28 clones as expression ELISA assay. The vNAR T1 has an approximately 3.5 times higher expression level than the other two isolated clones. Figure 1c shows that periplasmic extract of His-tagged vNAR T1 recognizes the rhTGF-β1 cytokine with statistical significance compared to 3% BSA used as negative control (P < 0.0001). The recognition ELISA assay was a preliminary test for screening between vNARs. However, in this preliminary assay, the T20 and T28 vNARs demonstrated no significant differences between cytokine or BSA recognition. Based on the expression and recognition ELISA assay results of periplasmic extract, only the vNAR T1 was expressed and purified for further analysis, eliminating the vNARs T20 and T28. The sequence of vNAR T1 is shown in Fig. 2a. The multiple sequence alignment (MSA) analysis was used to determine the CDR3 size of the vNAR T1, compared to other previously reported vNAR sequences (Fig. 2b) demonstrating a long and variable CDR3 (24 aa). Based on the vNAR T1 sequence, the dynamic molecular interaction of vNAR/TGF-β1 was analyzed using the PROCHECK server. In contrast, the quality of the optimal model for vNAR T1 (Fig. 2c) was evaluated using a Ramachandran plot (Fig. 2b). The statistics showed that 99 residues (96.1%) are in favored regions, and 4 residues (3.9%) are found in additional regions, demonstrating a good quality of the model.

#### Expression and purification of His-tagged vNAR T1

**Figure 3:**
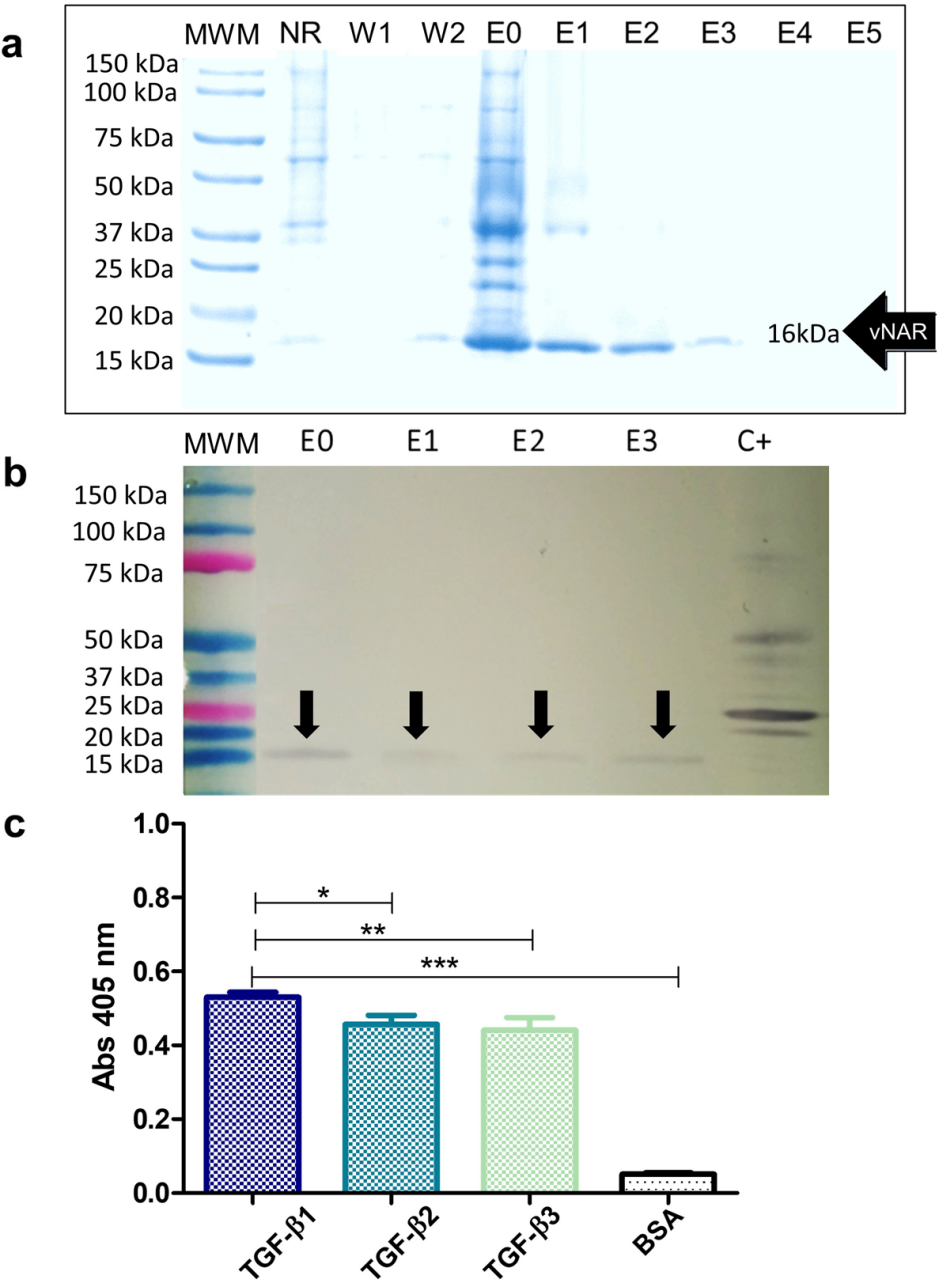
SDS-PAGE and western blot analysis for His-tagged vNAR T1 expression and binding to immobilized rhTGF-β isoforms by ELISA (**a**) 12% SDS-PAGE. MWM: Molecular weight marker, NR: Non-retained, W1-2: Wash solutions, E0-5: Elution fractions 0–5. (**b**) Western blotting. MWM: Molecular weight marker, E0-3: elution fractions, C + : Positive control (irrelevant non-related protein with a six His tag), (**c**) vNAR T1 binding to immobilized rhTGF-β isoforms by ELISA. Error bars represent standard deviation (s. d.). n = 3. ***P < 0.001, **P < 0.01, and *P < 0.05, n = 3. The purified his-tagged vNAT T1 recognizes three human TGF-β isoforms compared to 3% BSA (*** P < 0.001).

The vNAR T1 domain was expressed as a His-tagged protein, and it was extracted from the periplasmic space of *E. coli* ER2537 and purified by IMAC. The purified protein was visualized by Coomassie blue staining on SDS‐PAGE (Fig. 3a). Protein bands with apparent MW ~ 15–16 kDa were present in the *E. coli* protein extract and effectively purified. The His-tagged vNAR T1 was also detected by western blot (Fig. 3b) using a specific anti‐His-HRP antibody. The His-tagged vNAR T1 protein expressed in the periplasmic space of *E. coli* BL21 (DE3) cells showed a yield of 1.8 mg/L after purification.

#### vNAR T1 binding to immobilized rhTGF-β isoforms by ELISA

ELISA showed that His-tagged vNAR T1 recognized the three human isoforms of the rhTGF-β1-3 (Fig. 3c). The preference for the rhTGF-β1 isoform was observed (P < 0.005) and then for rhTGF-β2 and rhTGF-β3 (P < 0.01). However, no statistically significant difference was observed between the recognition of rhTGF-β2 and rhTGF-β3 isoforms.

#### SPR kinetic results of vNAR T1/TGF-β interaction

**Figure 4:**
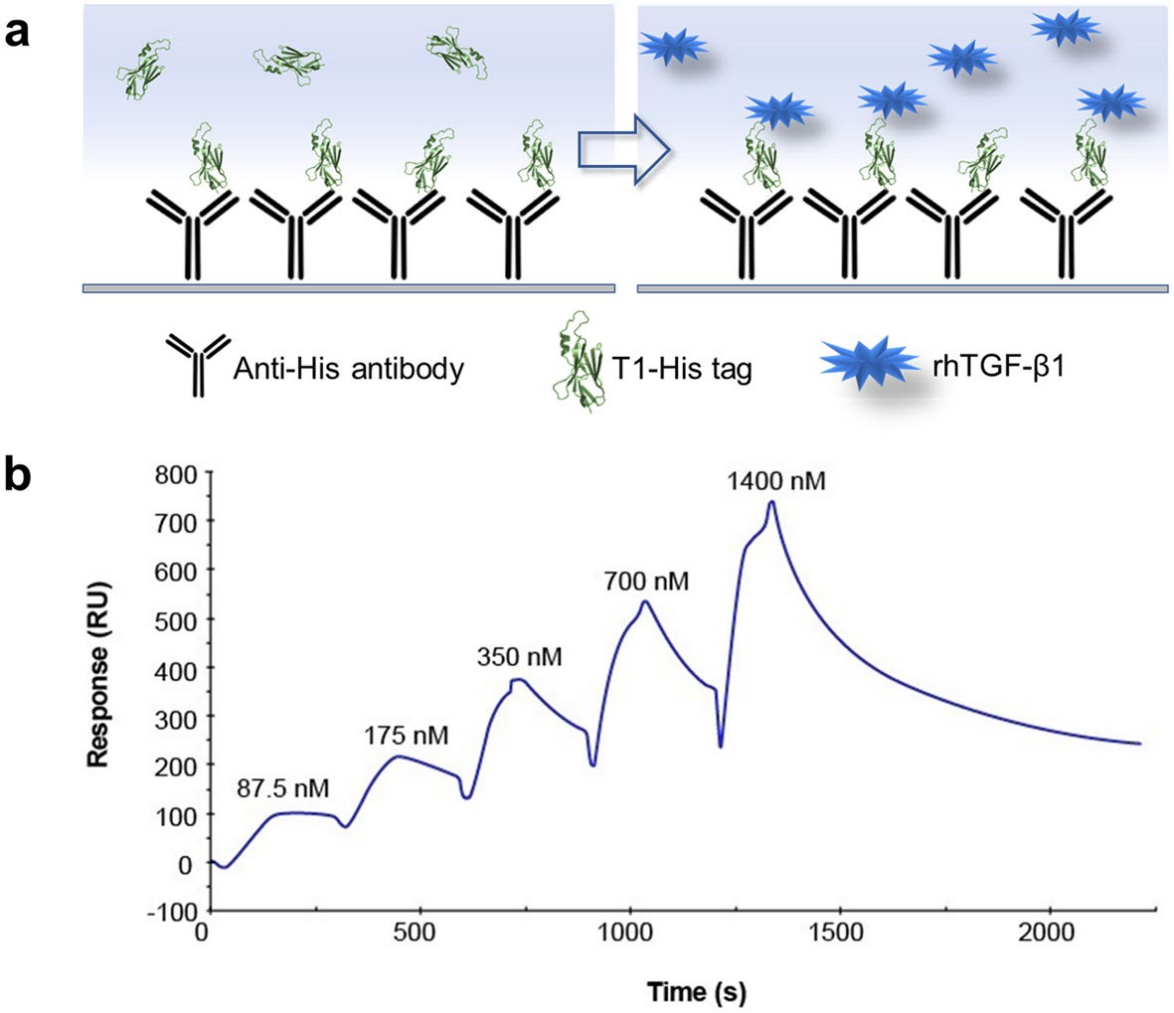
Surface Plasmon Resonance assay (SPR). (**a**) Schematic representation of SCK method. (**b**) Representation of Single-cycle kinetics (SCK) experiments of vNAR T1/rhTGF-β1. Sensorgram (blue dotted curve) of the response (resonance units, RU) versus time of the SCK by injecting five increasing concentrations (87.5 nM to 1,400 nM) of rhTGF-β1 over the vNAR T1 His-tag + Anti-His Antibody. The result represents the mean of three independent experiments.

The interaction kinetics of the His-tagged vNAR T1 and rhTGF-β1 complex was evaluated using an SCK method. Herein we propose to immobilize vNAR T1 via its His-tag allowing the CDRs to freely interact with the target (Fig. 4a). For this, an anti-His antibody (MyBioSource, MBS435072) was captured on the chip surface at > 12,000 RU. Figure 4b shows the sensorgram obtained from sequential injection of rhTGF-β1 at five gradually higher concentrations, ranging from 87.5 nM to 1,400 nM. The response signal increased after every injection and approached a steady-state value before the end of each injection, indicating the formation of the vNAR/TGF-β1 complex. Then, the buffer flowed over the complex, and the response signal decreased, indicating the dissociation of rhTGF-β1. The kinetic parameters of the vNAR/TGF-β1 interaction are presented in Table 1, where the vNAR T1 showed an affinity (*K*_*D*_) of 9.61 × 10^–8^ M as the mean of three independent assays.

**Table 1:** Kinetic measurements for the interaction of vNAR T1 and rhTGF-β1.

R_max_	Chi (× 10^3^)	*Ka* (× 10^+4^ M^-1^ s^-1^)	*Kd* (× 10^–4^ M^-1^ s^-1^)	*K*_*D*_ (10^–8^ M)
730.6	1.33	3.227	33.17	10.28
737.1	1.28	3.357	33.55	9.99
760.6	1.27	3.228	27.65	8.56
Mean	**1.29**	**3.27**	**31.34**	**9.61**

The association (Ka) and dissociation (Kd) rate constants and the equilibrium dissociation constant (K_D_) are shown. The results correspond to three independent assays.

#### In silico analysis of TGF-β and T1 interaction

**Figure 5:**
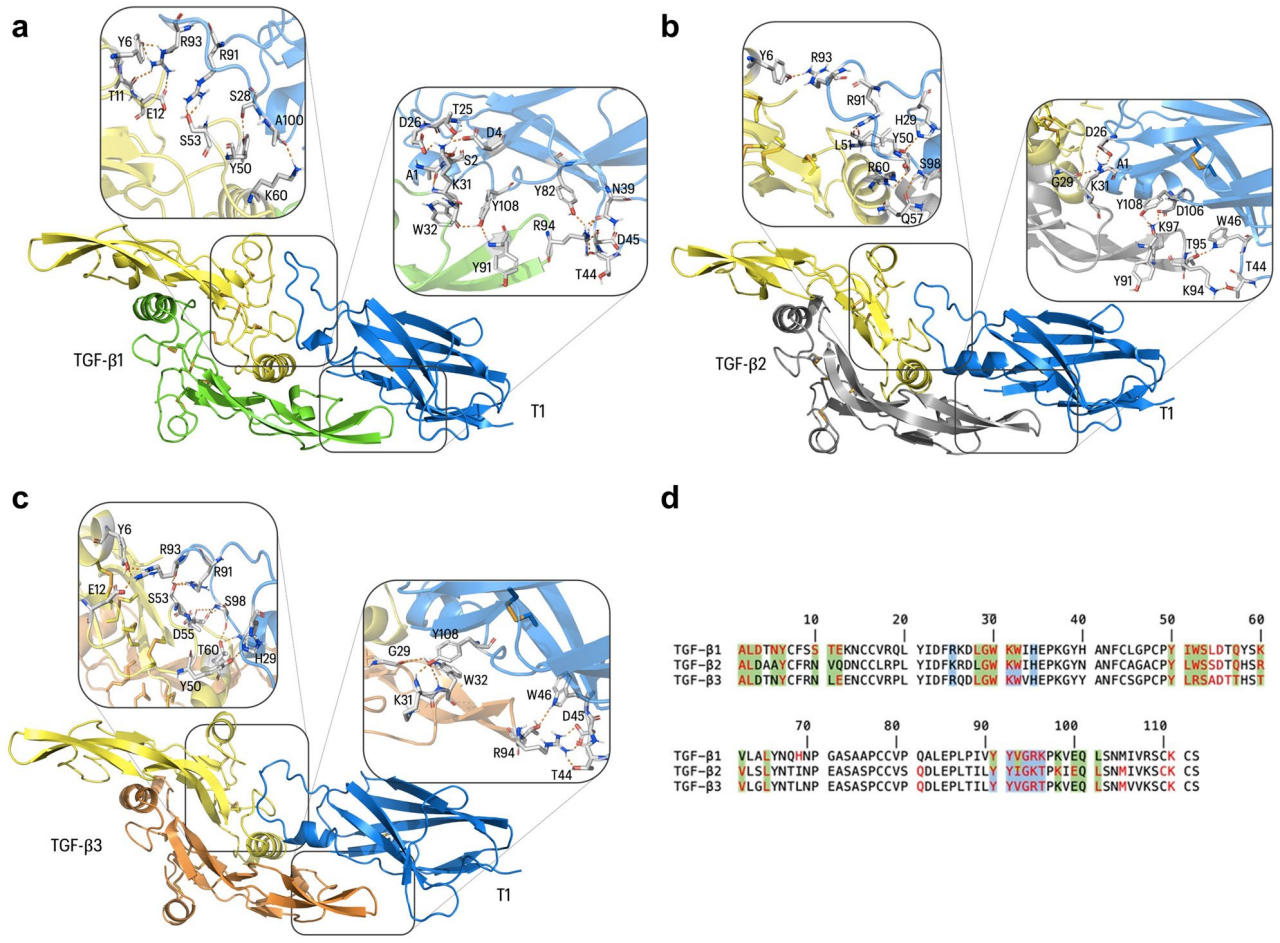
Structural analysis of vNAR T1 coupled to human TGF-β isoforms and competition binding assay. (**a**) vNAR T1 (blue) with TGF-β1 (yellow and green). The grey boxes show a detailed view of the interfacing residues (polar contacts as orange dash lines). (**b**) vNAR T1 (blue) interaction with TGF-β2 isoform (yellow and grey). (**c**) vNAR T1 (blue) interaction with TGF-β3 isoform (yellow and orange). All TGF-β isoform shows as a homodimeric soluble protein. (**d**) Sequence alignment of each TGF-β isoform, the interface residues of TGF-β1 interacting with the natural receptor type 2 (TβRII) are highlighted in blue, and the amino acids recognized for type 1 receptor (TβRI) were shown in green both (from the complex entry in PDB ID 3KFD). In red color are highlighted the residues of TGF-β amino acids recognized by the vNAR T1 domain.

The predicted interactions between the vNAR T1 and the TGF-β isoforms are shown in Fig. 5, including the specific amino acids. The vNAR interacts with the CDR3 and the HV2 regions with the homodimeric cytokine. In Supl. Table 1, where the vNAR T1 has the highest affinity with TGF-β1, scoring -27.20 REU (calculated by the Rosetta server), followed by TGF-β3 (-24.58 REU) and TGF-β2 (-18.35 REU). The interfacing residues were further evaluated with PDBePISA, showing 30 residues of TGF-β that interact with vNAR T1 (Fig. 5d). The CDR3 region of vNAR T1(86aa—QTIGRRKRGPLASLAAMMGSSDYY -109aa) interacts with amino acids that naturally bind to the native receptors for TGF-β^39^: 75% for the TβRI and 80% for the TβRII, surrounded by FR1 and the HV2 of vNAR T1. Sequence alignment of TGF-β isoforms (Fig. 5d) shows the interface residues of TGF-β1 interacting with the TβRII are highlighted in blue, and the amino acids recognized for TβRI were shown in green. In red are highlighted the amino acids of TGF-β recognized by the vNAR T1.

### In silico competition analysis of TGF-β/vNAR T1 and TGF-β/TβRII interaction

**Figure 6:**
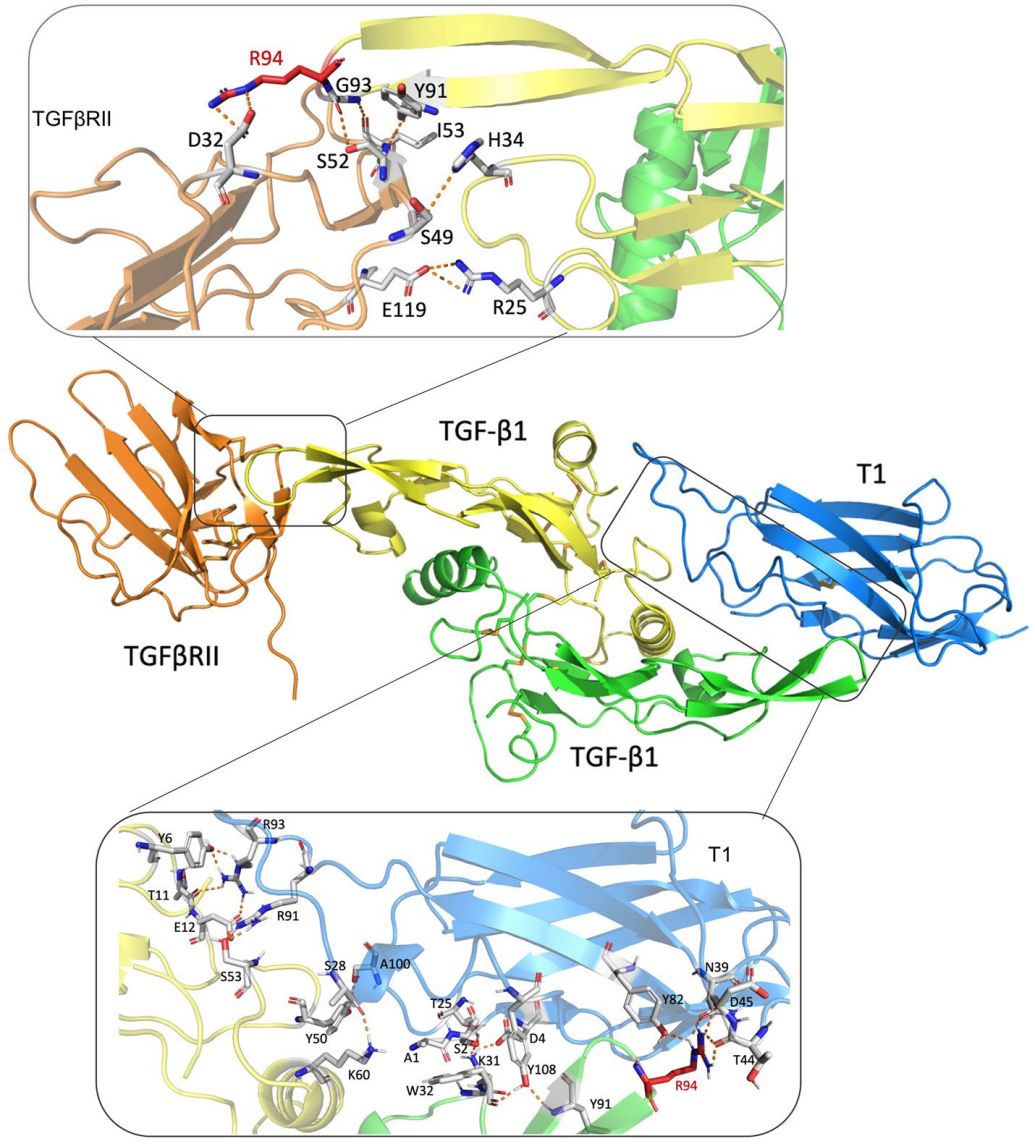
In silico competition binding assay of vNAR T1 and TβRII against TGF-β. The main amino acids of the complexes vNAR T1/TGF-β1 and TβRII/TGF-β1 are focused into de boxes and represented with amino acid abbreviations and numbers.

Figure 6 shows the comparison of amino acid interaction between the vNAR T1/TGF-β1 and TβRII/TGF-β1. The relevant amino acids are highlighted, and the TGF-β1 cytokine shows a homodimer (chain A and chain B). The vNAR CDR3 (blue) interacts with the cytokine chain A (yellow). Also, the HV2 region of vNAR T1 interacts with the chain B (green) of the cytokine. In contrast, the TβRII (orange) interacts only with the cytokine chain B (green). Table 2 shows the affinity determined between the vNAR T1 and each TGF-β isoform.

**Table 2:** Interaction analysis and in silico affinities of the TGF-β isoforms with vNAR T1.

	Chain	Cytokine sequence	vNAR T1/TGF-β affinity score (REU)
TGF-β1	*A*	**CPYIWSLDT**QYSK	− 27.44
	*B*	LYID**FRKDLGWKW**	
TGF-β2	*A*	AC**PYLWSSDTQ**HS	− 18.35
	*B*	IDFKRD**LGWKWI**H	
TGF-β3	*A*	Y**LRSADTTHSTVL**GL	− 24.58
	*B*	Y**YVGRTPKVEQLS**NM	

The amino acid sequence of each TGF-β isoform homodimer interacting with the vNAR T1 is highlighted in bold.

*REU* Rosetta energy units.

## Discussion

After three rounds of selection against rhTGF-β1 cytokine using the phage display technique, a vNAR domain was isolated from a non-immunized *H. francisci* shark library (Fig. 1). We selected a vNAR domain that is proficient in recognizing all three rhTGF-β isoforms. The sequence of vNAR T1 (Fig. 2) showed this is a type IV domain, according to Zielonka et al., based on the lack of non-canonical disulfide bridge that has been described for other vNAR types^30^. An exciting aspect of vNAR T1 is its extensive CDR3, composed of 24 amino acid residues (86aa—QTIGRRKRGPLASLAAMMGSSDYY -109aa). A reduced number of vNAR was reported with an extended CDR3 domain than the T1 domain, which is the case of the vNAR described by Leow et al. (2018), reporting a CDR3 of 24 residues of amino acids^40^ and the vNAR reported by Camacho-Villegas et al. with a CDR3 of 27 amino acids long^37^. The extensive CDR3 of vNARs is a considerable advantage acting like a long hairpin that favors access to cryptic epitopes. Furthermore, the long CDR3 tends to be associated with more amino acid interactions and protein/ligand interactions. Then, the pCOMb3X plasmid encoding for vNAR T1 was used for protein expression in a heterologous *E. coli* system (Fig. 3). Our results reveal the ability of vNAR T1 to specifically recognize the three human isoforms of TGF-β in an ELISA (Fig. 3c). The closest approach to this strategy is the sdAb isolated from a camel as describe by Henry et al.; however, it only recognizes TGF-β3^41^.

These results are relevant, considering the report of Yu et al., who reported a solid profibrotic effect of all three TGF-β isoforms and suggested that increasing TGF-β isoform concentrations can contribute to pathologic matrix accumulation in renal fibrosis. However, although TGF-β1 may be the principal mediator, the authors suggest that blocking all isoforms together may result in the best therapeutic effect^42^. Likewise, findings reported by Gupta et al. also appear to support this notion of an efficient therapy based on TGF-β neutralization, whether isoform-specific or pan-specific, providing a feasible option to deal with local immune resistance in cancer^43^. In this context, a selective mAb anti-latent TGF-β1^44^ was reported by Welsh et al. and could be evaluated in combination with a pan-specific anti-TGF-β domain described in this manuscript. The advantages of simultaneously modulating the biological effect of latent TGF-β1 (avoiding the cytokine activation in a proinflammatory microenvironment) and the soluble homodimer needs careful evaluation to avoid systemic adverse effects.

Most importantly, vNAR T1 recognition of hTGF-β1, -β2, and -β3 was consistent with the molecular docking results (Fig. 5). Nevertheless, in silico assays have shown that vNAR T1 presents the highest recognition for isoform TGF-β1, the predominant isoform circulating in mammals^45^. Highlight that TGF-β isoforms are similar in function and sequence and bind to the same receptors^46^. The predicted regions of amino acids that mediate the interaction of the vNAR/TGF-β complex were detected. The results showed that vNAR T1 recognizes amino acid residues of TGF-β1 (Arg 94, Ile 51, Gln 57, and Lys 60), which are necessary for recognition by type I and II surface receptors^39^. These outcomes suggest that vNAR T1 could recognize and neutralize the active form of the TGF-β by blocking the formation of the assemble with TβRI and TβRII obstructing the binding of the TβRI2-TβRII2 heterotetramer, which is necessary for the intracellular TGF-β signaling^47–49^. Even the commercial mAbs Fresolimumab recognize the same amino acids in the cytokine as the TβRI and TβRII^50^. Thus, evidence sustains our proposal that the vNAR T1 can prevent the binding of TGF-β with both receptors.

Our results suggest a similar mechanism of action compared to chimeric proteins comprising the ligand-interacting ectodomains of receptors fused with the human IgG1 Fc domain. Yung et al. informed the inhibition of biological activities of TGF-β1 and TGF-β3 using a soluble TβRII receptor extracellular domain expressed as an immunoglobulin-Fc fusion protein (TβRII-Fc)^51^. Takahashi et al. developed a chimeric protein, TβRI-TβRII-Fc, although this chimeric protein interacted with all TGF-β isoforms and overcame the problems of the effective concentration of both ligand traps and differences in the half-lives of TGF-β receptor types. The effectiveness of this chimeric protein suggests that the TβRI-TβRII-Fc is a promising tool for developing effective therapies based on inhibiting TGF-β signals. However, it is crucial to keep in mind the high molecular weight of this chimeric protein of  ~100 kDa^16^. In that sense, the vNAR T1 could overcome the size and complexity of the protein, being 10 times smaller than a whole IgG and six times smaller than the chimeric protein TβRI-TβRII-Fc.

The equilibrium dissociation constant (*K*_*D*_) of the vNAR T1/TGF-β1 complex was determined by SCK and showed an affinity to rhTGF-β1 of 9.61 × 10^–8^ M (96.1 nM). Nevertheless, further analysis is required to study the affinity of the TGF-β2 and TGF-β3 isoforms. vNAR T1 has a competitive binding affinity (lower *K*_*D*_) to the TGF-β1 isoform. Bedinger et al. isolated and characterized human antibodies that bind and neutralize different isoforms of TGF-β, where the affinity of a pan-specific antibody XPA.42.068 is 59 pM and for two versions of affinity-maturated antibodies (i.e., XPA.42.681 and XPA.42.089) were ≤ 10 pM for each isoform. Also, they report the affinity characterization of the mAb 1D11 (Invitrogen, MA5-23795), with 72, 170, and 78 pM against the TGF- β1, -β2, and -β3 isoforms, respectively^52^. Our vNAR T1 has less affinity, possibly associated with the monovalency (one TGF-β molecule with one vNAR T1) compared to the IgGs bivalency (two TGF-β with one IgG). Further, Sepehri et al. express that a current challenge of mAbs is the improvement of tissue penetration, which is considerably limited by their large size (150 kDa) even though mAbs reported have high specificity and affinity^53^. The vNAR T1 (15–16 kDa) overcome this limitation under this context. Therefore, an antibody domain that can recognize the three TGF-β isoforms can be advantageous and relevant from a therapeutic perspective^1^, Yang et al. reported a 75% metastasis suppression in 12 breast cancer models when Fresolimumab, a pan-specific TGF-β, was administrated^18^. Greco et al., reported tumor regression and long CD8^+^ antitumor immunity when combinatory immunotherapy was used (anti-PDL-1 and a modified Fresolimumab) in a preclinical test^54^. vNAR T1 can highly bind to an excess of TGF-β concentration in a tissue microenvironment (i.e., fibrosis or cancer) and cleared quickly by glomerular filtration^55^, modulating the biological effect; this supposes a rapid diminution of TGF-β concentration and could use as part of immunotherapy in combination with chemo drugs or with an immune checkpoint agent specifically for cancer treatment. This novel hypothetical approach could avoid damage related to completely neutralizing TGF-β pleiotropic function in normal tissues. Huang et al. suggest that long-term blockage of this cytokine causes adverse effects such as chronic inflammation or inflammatory lesions in heart valves^56^; avoiding the use of vNAR T1 in chronic disease treatment could reduce the potential damage in normal tissues. Nevertheless, more assays are required to elucidate the mechanism and safety of this approximation.

However, substantial efforts to improve the vNARs pharmacokinetic (PK) properties are explored, such as the systemic half-life^55^, i.e., increasing the size conjugated with HAS^57^ or Fc region, to prevent glomerular clearance. For the vNAR T1, those are other opportunities that could be explored.

Table 2 identifies the amino acids of the TGF-β isoforms recognized by vNAR T1. The amino acids of TGF-β that coincide in the interactions with vNAR T1 and TβRII (highlighted in red) are identified. The interaction takes place in the same region for the three isoforms. Further, these results suggest that vNAR T1 may block receptor binding. The in silico affinity is technically the same between vNAR T1/TGF-β1 (− 27.20 REU) and TGFβRII/TGF-β1 (− 27.44 REU). Therefore the vNAR T1 is potentially a pan-specific neutralizing agent for TGF-β isoforms. In the interaction of the TGF-β3 isoform with vNAR T1, we found an in silico affinity (− 24.58 REU) like the affinity for TGF-β1. However, in the interaction of the TGF-β2 isoform with vNAR T1 we found an in silico affinity was minor (− 18.35 REU). Attributed to amino acid residues substitution R25, V92, R94, which have been described as responsible for a high affinity between TGF-β1/TGFβRII and TGFβ3/TGFβRII^58^. Determining the affinity of the vNAR T1/TGF-β2 and vNAR T1/TGF-β3 complexes using SKC is considered a perspective.

Also, future studies must evaluate the immunological functions of vNAR T1 compared with conventional mAbs. Furthermore, the therapeutic combination of vNAR T1 with other vNARs with the potential to act as neutralizing domains against emerging variants of SARS-CoV-2 could be considered^59^ or in the severe coronavirus disease 2019 (COVID-19)^9^. Moreover, the detection of TGF-β has been proposed for diagnosis and prognostic stratification^1^. In this sense, the vNAR T1 could also be used as an element of TGF-β detection in immunoassays.

## Conclusions

Several reports prove the possibility of isolating vNAR from immunized and non-immunized sharks. These domains maintain their recognition ability, high affinity, and selectivity for the molecular target screened by the phage display technique. Panning of a library from a non-immunized *H. francisci* shark library resulted in the isolation of vNAR T1, with pan-specific recognition of the three TGF-β isoforms as demonstrated in vitro and in silico analysis.

Also, we successfully evaluated a vNAR binding in vitro by SRP for the first time to determine characteristics such as kinetics and affinity and in silico by molecular docking. Likewise, our interaction analysis results indicate that vNAR T1 recognizes amino acids involved in the interaction of TGF-β and the TGF- β Type I and II receptors that are crucial for the cellular signaling of TGF-β. As such, the pan-specific vNAR T1 can be seen as a potential therapeutic agent capable of modulating TGF-β signaling in diseases such as cancer and fibrosis.

## Material and Methods

### Selection of a vNAR antibody isolated from a non-immune library

A phage display was performed to select a specific vNAR, starting with a naive vNAR library of *H. francisci* shark in the pCOMb3X plasmid previously generated^37^. After reamplification, phages were obtained against rhTGF-β cytokine (Peprotech, 100-21) resuspended in 10 mM citric acid, pH 3.0, according to manufacturer instructions. Two wells of a 96-well plate coated with rhTGF-β (5 μg/mL) and incubated for 1 h at 37 °C. Wells were blocked with 150 μL of PBS-BSA 3% for 1 h at 37 °C. Then, 50 μL of phages were added and incubated at 37 °C for 1 h. Then, the washing steps are gradually increased to 7 for round 1, 14 for round 2, and 21 for round 3 to increase the stringency. These washes raise 150 μL of TBS-Tween 0.05% (TBST) per well five times and are allowed to stand 5 min between each wash. After the wash rounds, 50 μL of trypsin 10 μg/mL was added in 1% BSA, followed by 30 min at 37 °C incubation. The wells were washed by raising the solution volume vigorously ten times and using the eluted phages to infect a culture of 2 mL of *E. coli* strain ER2537 (OD_600nm_ = 1), followed by incubation of 15 min at room temperature. Finally, transferring the culture to a 50 mL tube containing 6 mL SB medium and 1.6 μL carbenicillin (100 mg/mL, Sigma, C1389) and incubated for 1 h at 37 °C at 250 pm. The output titration count was obtained with 2 μL of the initial 8 mL culture and diluted in 200 μL of SB medium, plating 10 μL and 100 μL in LB carbenicillin plates. To the input result, a culture of 2 mL of ER2537 cells was grown at an OD_600nm_ = 1. Then 50 μL was infected with 1 μL of a 1:10^–8^ dilution of phages obtained after each panning round and incubated for 15 min at room temperature, finally plated onto LB agar plates with carbenicillin (100 μg/mL). The plates were incubated overnight (ON) at 37 °C. After incubation in standard conditions, the input and output titers were obtained by multiplying the number of colonies by the culture volume and dividing by the plating volume^38^.

After the 1 h incubation of the 8 mL culture, 2.4 μL of carbenicillin (100 mg/mL) was added, and the tube was incubated for another hour and transferred to a 500 mL flask. Next, 1 mL of helper phage VCSM13 phage VCSM13 (Stratagene, 200251), 91 mL of SB medium, and 46 μL of carbenicillin (100 mg/mL) were added to the flask and incubated for 2 h at 37 °C and 250 rpm. Then, 140 μL of kanamycin (Sigma, 60615) was added at 50 mg/mL and incubated for 12 to 16 h. This protocol was repeated in each round, except the next rounds used only one well with an immobilized cytokine and increased washed steps of 7, 14, and 21. Finally, a colony PCR screening selects clones with the vNAR sequence.

### vNAR expression and purification

The positive pCOMB3X plasmid containing the His-tagged vNAR sequence was transformed into *E. coli* BL21 (DE3) cells. An isolated colony was grown in 3 mL of LB medium supplemented with 100 μg/mL ampicillin and incubated for 12 h at 37 °C and 250 rpm. The overnight culture was added to 250 mL of fresh medium with the same antibiotic concentration and further cultured under the same culture conditions. Once the culture reached an OD_600nm_ = 0.7, expression was induced by adding 0.5 mL of IPTG 0.5 M (Sigma, I5502), followed by an incubation of 5 h at 37 °C at 250 rpm. The vNAR was isolated from periplasmic space by osmotic shock and used to make the first screening for expression and recognition ELISA assays. The periplasmic extract of the clone that met both requirements was filtered through a 0.2 μm and purified by IMAC (Thermo Fisher Scientific, 88221). The NiNTA column was equilibrated with wash buffer (20 mM imidazole, 50 mM NaPO_4_, 300 mM NaCl, pH 8.0), the periplasmic extract was loaded using a syringe at 1 mL/min constant flux and then washed with 10 mL of wash buffer. Bound His-tagged vNAR was eluted with 5 mL of elution buffer (250 mM imidazole, 50 mM NaPO_4_, 300 mM NaCl, pH 8.0) and collected in 1 mL fractions. Before proceeding with the ELISA assay, the fractions containing the vNAR were dialyzed extensively against 0.5X PBS. Fractions were quantified using the Micro BCA kit (Thermo Scientific, 23235) and analyzed by SDS-PAGE and western blotting. An SDS-TRICINE-PAGE was run at 120 V for 45 min and stained with Coomassie brilliant blue with the Precision plus protein™ Dual-color standards (BioRad, 1610394) as a molecular protein marker. For the western blot analysis, proteins were transferred from the gel to a nitrocellulose membrane for 1 h at 200 mA using a Trans-blot semi-dry electrophoretic transfer cell (BioRad, 1703940). The membrane was blocked with 3% BSA-PBS for 1 h at room temperature with constant agitation. After discarding the blocking solution, anti-His-HRP (Roche, 11965085001) diluted 1:1,000 in 1% BSA-PBS was added, followed by incubation for 1 h at 37 °C. The membrane was then washed thrice with PBST for 2 min, and proteins were made visible using an HRP color development reagent (BioRad, 1706534).

### Reactivity of vNAR against rhTGF-β isoforms by ELISA

An ELISA assay was performed by adding 250 ng of rhTGF-β1 (Peprotech, 100–21, resuspended in 10 mM citric acid pH3.0) and rhTGF-β2 isoforms (Peprotech, 100–35, resuspended H_2_O) per well to analyze if the vNAR antibody recognized rhTGF isoforms. For rhTGF-β3 Isoform (Peprotech, 100-36E resuspended in 10 mM citric acid, pH 3.0), 125 ng/well was used, considering the initial cytokine concentration stock. The final volume for all cytokines was 50 μL in wells. The plate was incubated for 2 h at 37 °C. The solution was discarded and then blocked with 150 μL 3% BSA-PBS for 1 h at 37 °C. Then discarded, after 250 ng of the vNAR T1 was added to each well and incubated for 2 h at 37 °C. The wells were washed three times with phosphate-buffered saline Tween (PBST) solution, after which 50 μL of anti-HA-HRP antibody (Roche, 12013819001) diluted 1:1,000 in 1% BSA-PBS solution was added, followed by a 2 h incubation at 37 °C. After three washes with PBST, 50 μL of TMB ELISA reagent (Thermo Scientific, T0440) was added per well. The plate was incubated at 37 °C for 10 min and analyzed at 405 nm on an xMark microplate absorbance spectrophotometer (BioRad, 1681150). The negative control consists of 3% BSA. All assays are in triplicate.

### Surface plasmon resonance (SPR)

The equilibrium dissociation constant (*K*_*D*_) was determined using Biacore X100 (GE Healthcare) equipment. An anti-histidine IgG antibody was immobilized onto a CM5 chip (GE HealthCare, BR100012) at 50 μg/mL in 10 mM sodium acetate (pH 4.5) and immobilized at a flow rate of 10 μL/min using amine-coupling chemistry according to the manufacturer’s instructions until to standardize a > 12,000-RU surfaces (His Capture Kit, GE Healthcare, 28995056). The His-tagged vNAR T1 was captured and crosslinked to the anti-His IgG antibody previously immobilized on one surface; 1 μg/mL vNAR T1 was injected across the surface for 120 s at a flow rate of 10 μL/min. For the kinetic experiments, we used the method initially proposed by Karlsson et al., called Single-Cycle Kinetics (SCK), as a faster method than the classical SPR. SCK requires fewer regeneration steps and reduces costs^60,61^, as it injects increasing concentrations of the ligand in the solution, with only one regeneration step performed at the end of the complete binding cycle^62^. rhTGF-β1 samples were prepared using a two-fold increased concentration gradient (0.08 to 1.4 μM) in HBS-EP + buffer (GE Healthcare, BR100826). Two injections of HBS-EP + running buffer were performed along with the samples to compensate for systemic effects by double referencing. Kinetic rate constants were derived from double-referenced sensorgrams by global fitting. Local Rmax was used to consider the slight loss of surface activity and not adjust for bulk changes in the refractive index. Equilibrium dissociation constants (*K*_*D*_) were derived from plots showing the concentration-dependent steady-state binding of rhTGF-β1 to vNAR T1 by a nonlinear curve fitting to a 1:1 interaction model using Biacore X100 control software 2.0.1. The sensorgrams were fitted using the Langmuir 1:1 binding model to extract the kinetic parameters of the vNAR/TGF-β1 interaction.

### In silico assays: homology modeling and molecular dynamics

The three-dimensional (3D) structure of vNAR T1 was predicted by homology-based modeling using MODELLER v.9.16^63^. Nanoscale Molecular Dynamics (NAMD) software^64^ was used to refine the 3D structure of the vNAR T1. The results were visualized and analyzed using MacPyMOL (v2.2.2 license #27614) and Visual Molecular Dynamics (VMD)^65^. The quality of the vNAR T1 structure was evaluated by a Ramachandran plot using the PROCHECK server (https://saves.mbi.ucla.edu/)^66^. Molecular dynamics were performed by simulated annealing strategy according to the previously described by Cabanillas et al.^67^.

### Molecular docking vNAR T1/TGF-β isoforms or TGF-β/TGFβRII

A protein–protein docking protocol was performed to predict the potential binding site of the vNAR T1 to TGF-β isoforms using the ClusPro web tool (https://cluspro.bu.edu/)^68^. The models were obtained with MODELLER v.9.16^63^ and refined with NAMD with 50 ns of contact. The model with extended time in the dynamic in silico assay was selected as the most thermostable. The vNAR-TGF-β complex (all three isoforms) with good electrostatics and desolvation-free energies were selected. The protein–protein interaction regions were predicted using Peptiderive, located on the ROSIE server (https://rosie.graylab.jhu.edu/peptiderive/). Default settings and the plots with the predicted protein–protein interactions were ranked according to the Rosetta Energy Units (REU)^68,69^. The server PDBePISA (http://www.ebi.ac.uk/pdbe/prot_int/pistart.html)^69^ was used to individually analyze interfacing residues between hTGF-β1(PDB ID 1KLA), TGF-β2 (PDB ID 2TGI), TGF-β3 (PDB ID 1KLA) and the vNAR T1 or with TGF-βRII receptor (PDB ID 3KFD).

### Statistical analysis

The vNAR recombinant expression ELISA was compared with Two-way ANOVA followed by Tukey’s multiple comparison test. For comparison of vNAR binding ELISA against rhTGF-β isoforms, a One-Way ANOVA followed by Tukey’s post hoc test was performed. A P < 0.05 value was considered for all data and indicated in all figure legends. Values are presented as means ± standard deviation (s.d.). All analyses were performed in the PRISMA Graph pad software.

## Supplementary Information

Supplementary Information.

## Data Availability

All data generated or analyzed during this study are included in this published article.
