# Supplementary material for: First pan-specific vNAR against human TGF-β as a potential therapeutic application: in silico modeling assessment
Source: Sci Rep. 2023 Mar 3;13:3596. doi: 10.1038/s41598-023-30623-x (PMC9982792; doi:10.1038/s41598-023-30623-x)
Supplement: Supplementary file 1 — Supplementary Information. [file 41598_2023_30623_MOESM1_ESM.docx]

**Supplementary Table 1.** Molecular docking and vNAR T1 amino acid sequence that interacts with each TGF-β isoform.

| **Molecular docking TGF-β1 /vNAR T1** | | | | | | | | |
| --- | --- | --- | --- | --- | --- | --- | --- | --- |
| TGF-β1 | | vNAR T1 | | Score  [REU] | | TGF-β1 Sequence | | vNAR T1 Sequence |
| A | | **T** | | -15.16 | | CPYIWSLDTQYSK | | **RKRGPLASLAAMM** |
| B | | **T** | | -12.04 | | LYIDFRKDLGWKW | | **AAMMGSSDYY** |
| Total score | |  | | **-27.20** | |  | |  |
|  | | | | | | | | |
| **Molecular docking TGF-β2 /vNAR T1** | | | | | | | | |
| TGF-β2 | | vNAR T1 | | Score  [REU] | | TGF-β2 Sequence | | vNAR T1 Sequence |
| A | | **T** | | -14.01 | | ACPYLWSSDTQHS | | **MMGSSDYY**GAGTV |
| B | | **T** | | -4.34 | | IDFKRDLGWKWIH | | **NNPG**STDWE |
| Total score | |  | | **-18.35** | |  | |  |
|  | | | | | | | | |
| **Molecular docking TGF-β3 /vNAR T1** | | | | | | | | |
| TGF-β3 | vNAR T1 | | Score  [REU] | | TGF-β3 Sequence | | vNAR T1 Sequence | |
| A | **T** | | -12.51 | | YLRSADTTHSTVLGL | | **RRKRGPLASLAAMMG** | |
| B | **T** | | -12.07 | | YYVGRTPKVEQLSNM | | **SLAAMMGSSDYY**GAG | |
| Total score |  | | **-24.58** | |  | |  | |

In silico affinities determined for the interaction of **vNAR T1 amino acid sequence that interacts with each** TGF-β isoform. The amino acid sequence of each TGF-β isoform that interacts with the vNAR is highlighted in red. The vNAR T1 amino acid sequence in CDR3 that interact with the cytokine is highlighted in blue. The vNAR T1 amino acid sequence in the HRV2 region that interacts with the cytokine is highlighted in purple. The in silico total score (REU) is calculated individually for each cytokine chain and summed up for the total score.
